# Effects of knocking out three anthocyanin modification genes on the blue pigmentation of gentian flowers

**DOI:** 10.1038/s41598-019-51808-3

**Published:** 2019-11-01

**Authors:** Keisuke Tasaki, Atsumi Higuchi, Aiko Watanabe, Nobuhiro Sasaki, Masahiro Nishihara

**Affiliations:** 1 Iwate Biotechnology Research Center, 22-174-4 Narita, Kitakami, Iwate 024-0003 Japan; 2 Present Address: Tokyo University of Agriculture, Atsugi, Kanagawa 243-0034 Japan; 3 Present Address: Toyo University, 1-1-1 Izumino, Itakura-machi, Ora-gun, Gunma 374-0193 Japan

**Keywords:** Molecular engineering in plants, Secondary metabolism

## Abstract

Genome editing by the CRISPR/Cas9 system has recently been used to produce gene knockout lines in many plant species. We applied this system to analyze Japanese gentian plants that produce blue flowers because of the accumulation of a polyacylated anthocyanin, gentiodelphin. Mutant lines in which anthocyanin modification genes were knocked out were examined to assess the contribution of each gene to the blue pigmentation of flowers. The targeted genes encoded anthocyanin 5-*O*-glycosyltransferase (*Gt*5*GT*), anthocyanin 3′-*O*-glycosyltransferase (*Gt3*′*GT*), and anthocyanin 5/3′-aromatic acyltransferase (*Gt*5*/3*′*AT*). The *Gt5GT* knockout lines accumulated delphinidin 3G, whereas the *Gt3*′*GT* knockout lines accumulated delphinidin 3G-5CafG as the major flower pigment. Knocking out *Gt5/3*′*AT* resulted in the accumulation of delphinidin 3G-5G-3′G and delphinidin 3G-5G as the primary and secondary pigments, respectively. These results indicated the existence of two pathways mediating the modification of delphinidin 3G-5G in flowers, with one involving a glycosylation by 3′GT and the other involving an acylation by 5/3′AT. The *Gt5GT*, *Gt3*′*GT*, and *Gt5/3*′*AT* transformants produced pale red violet, dull pink, and pale mauve flowers, respectively, unlike the vivid blue flowers of wild-type plants. Thus, the glycosylation and subsequent acylation of the 3′-hydroxy group of the B-ring in delphinidin aglycone is essential for the development of blue gentian flowers.

## Introduction

Flavonoids, carotenoids, betalains, and chlorophylls, which are the four major pigments in higher plants^1^, are distributed in almost all plant organs, including flowers, fruits, seeds, roots, stems, and leaves, wherein they play important roles in plant development, propagation and survival. Specifically, the coloration of flowers and fruits that helps attract pollinators and seed dispersers is crucial for plant reproduction under natural conditions. Among these pigments, anthocyanins, belonging to the large flavonoid family, produce diverse colors such as red, purple, and blue^2^. This diversity is mostly due to modifications of the anthocyanidin skeleton with sugars and aromatic acids as well as the number and position of hydroxyl and methoxyl groups attached to the basic anthocyanidin aglycone molecule. More than 100 kinds of polyacylated anthocyanins with several aromatic acids have been isolated^3^. The thorough characterization of the flavonoid biosynthetic pathway in higher plants has revealed that the genes responsible for the biosynthesis of anthocyanidin aglycone are highly conserved among plant species^4^. However, the genes responsible for the subsequent modifications after the initial 3-*O*-glycosylation are highly diverse among plant species and plant organs. The accumulated anthocyanins vary even within the same plant species, with flowers, in particular, producing various anthocyanin types.

Blue-flowered Japanese gentians accumulate a polyacylated anthocyanin, gentiodelphin [delphinidin 3-*O*-*β*-d-glucosyl-5-*O*-(6-*O*-caffeoyl-*β*-d-glucoside)-3′-*O*-(6-*O*-caffeoyl-*β*-d-glucoside)], as a major pigment^5,6^. All of the flavonoid biosynthetic structural genes that contribute to gentiodelphin biosynthesis have been identified^7,8^. Furthermore, several transcription factor genes, such as *MYB* and *bHLH*, regulating flavonoid biosynthesis have been isolated and characterized^9–11^. For example, a previous transient expression assay demonstrated that GtMYB3, which belongs to the R2R3MYB family, binds to the promoters of the gentian *F3*′*5*′*H* and *5/3*′*AT* (5,3′-aromatic acyltransferase) genes to upregulate expression^9^. Additionally, an analysis of the expression of *GtMYB3* with the SRDX motif, which is a dominant negative regulatory domain, confirmed that GtMYB3 is also a positive regulator of several downstream flavonoid biosynthetic genes in gentian flowers^12^.

**Figure 1:**
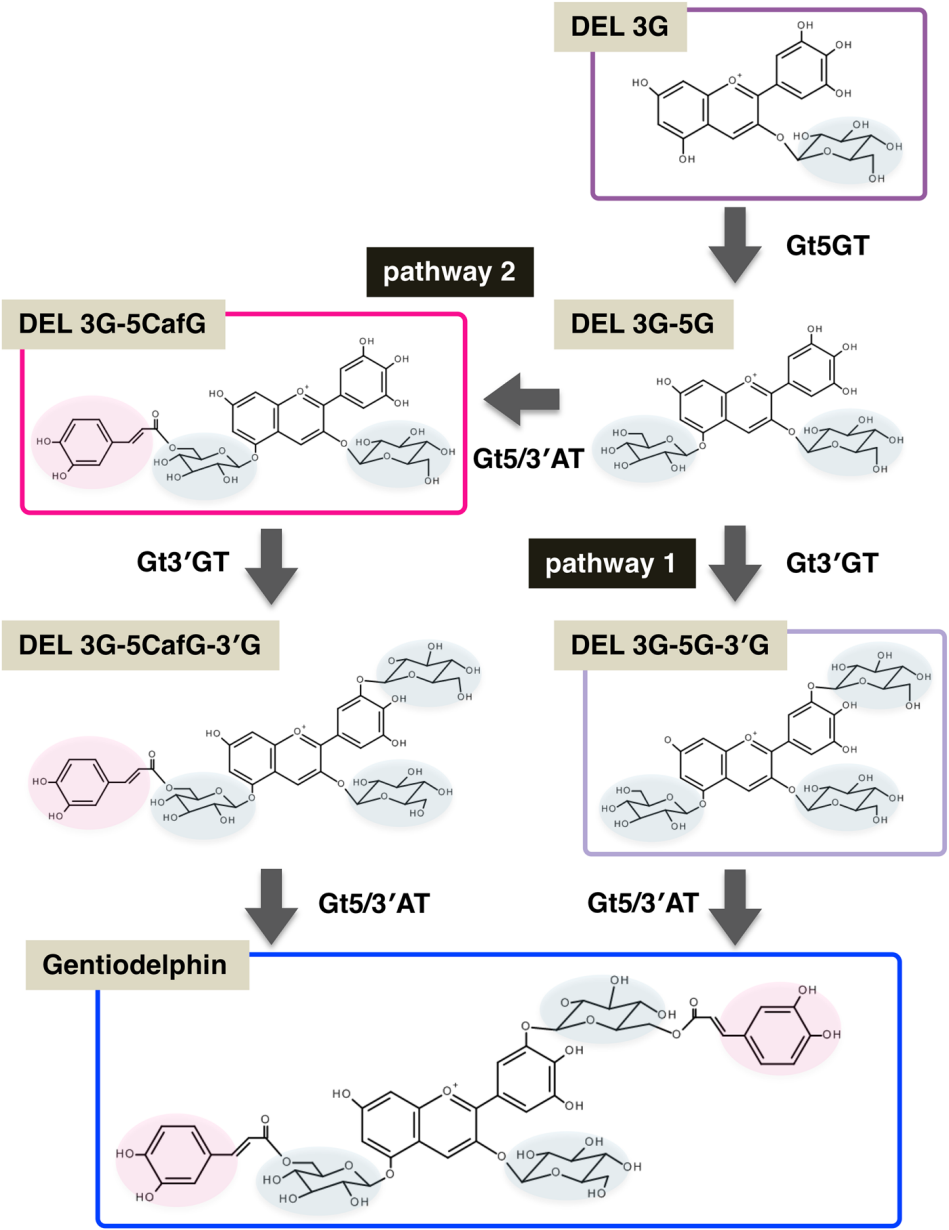
Gentiodelphin biosynthetic pathway. Gt5GT, UDP-glucose:anthocyanin 5-*O*-glucosyltransferase; Gt3′GT, UDP-glucose:anthocyanin 3′-*O*-glucosyltransferase; Gt5/3′AT, anthocyanin 5,3′-aromatic acyltransferase.

The conversion of delphinidin to gentiodelphin involves four modification steps (Fig. 1). Namely, delphinidin 3-*O*-glucoside is formed by the glycosylation of delphinidin via Gt3GT activity. Thereafter, additional sugar and acyl modifications are catalyzed by three key enzymes, including two glycosyltransferases, Gt5GT and Gt3′GT, and one acyl transferase, Gt5/3′AT, to produce gentiodelphin (Fig. 1). Regarding the polyacylated gentiodelphin structure, *in vitro* biochemical analyses suggested that the glycosylation and subsequent acylation of the 3′-hydroxy group of the B-ring of delphinidin aglycone are important for the stabilization and intramolecular sandwich-type stacking of two gentiodelphin caffeic acids, ultimately leading to a more intense blue coloration^5,13–15^. However, the functions of these modification enzymes *in vivo* have not been comprehensively characterized in gentian because of a lack of available lines in which these genes have been mutated. We previously generated mutant lines in which the expression levels of one (*Gt5/3*′*AT*) or two (*Gt5/3*′*AT* and *GtF3*′*5*′*H*) genes were knocked down by RNA interference (RNAi), and revealed that two transgenic lines exhibited abnormal flower coloration due to the decreased acylation of anthocyanins^16^. However, the effects were somewhat ambiguous because of the insufficient suppression of the target genes. The expression of the target genes was only downregulated rather than eliminated in the RNAi lines, and we were unable to analyze the effects of the complete inhibition of the expression of the anthocyanin modification genes. Furthermore, there are no available reports describing the suppression of *Gt5GT* and *Gt3*′*GT* expression.

Genome editing by the clustered regularly interspaced short palindromic repeats (CRISPR)/CRISPR-associated protein 9 (Cas9) system has recently been used to target specific genes in various higher plants, including ornamental plants, such as *DFR* in Japanese morning glory (*Ipomoea nil*)^17^, *F3H* in torenia^18^, and *PDS* in gentian^19^. Therefore, it is now possible to produce and analyze gene knockout mutant lines targeting genes of interest even in horticultural plant species.

In this study, we used the CRISPR/Cas9 system to target three anthocyanin modification genes (*Gt5GT*, *Gt3*′*GT*, and *Gt5/3*′*AT*) and analyze the effects of the knockout of these genes on the pigmentation of gentian flowers. We generated genome-edited lines in which the three targeted anthocyanin modification genes were knocked out and subsequently evaluated the effects on flower color. We confirmed the genome was accurately edited by next-generation sequencing (NGS) before examining flower colors with a spectrophotometer and by a compositional analysis of anthocyanin pigments via HPLC. Our results verified the utility of CRISPR/Cas9-based genome editing and the importance of anthocyanin modification genes for the blue coloration of gentian flowers.

## Results

### Production of genome-edited gentian plants

**Figure 2:**
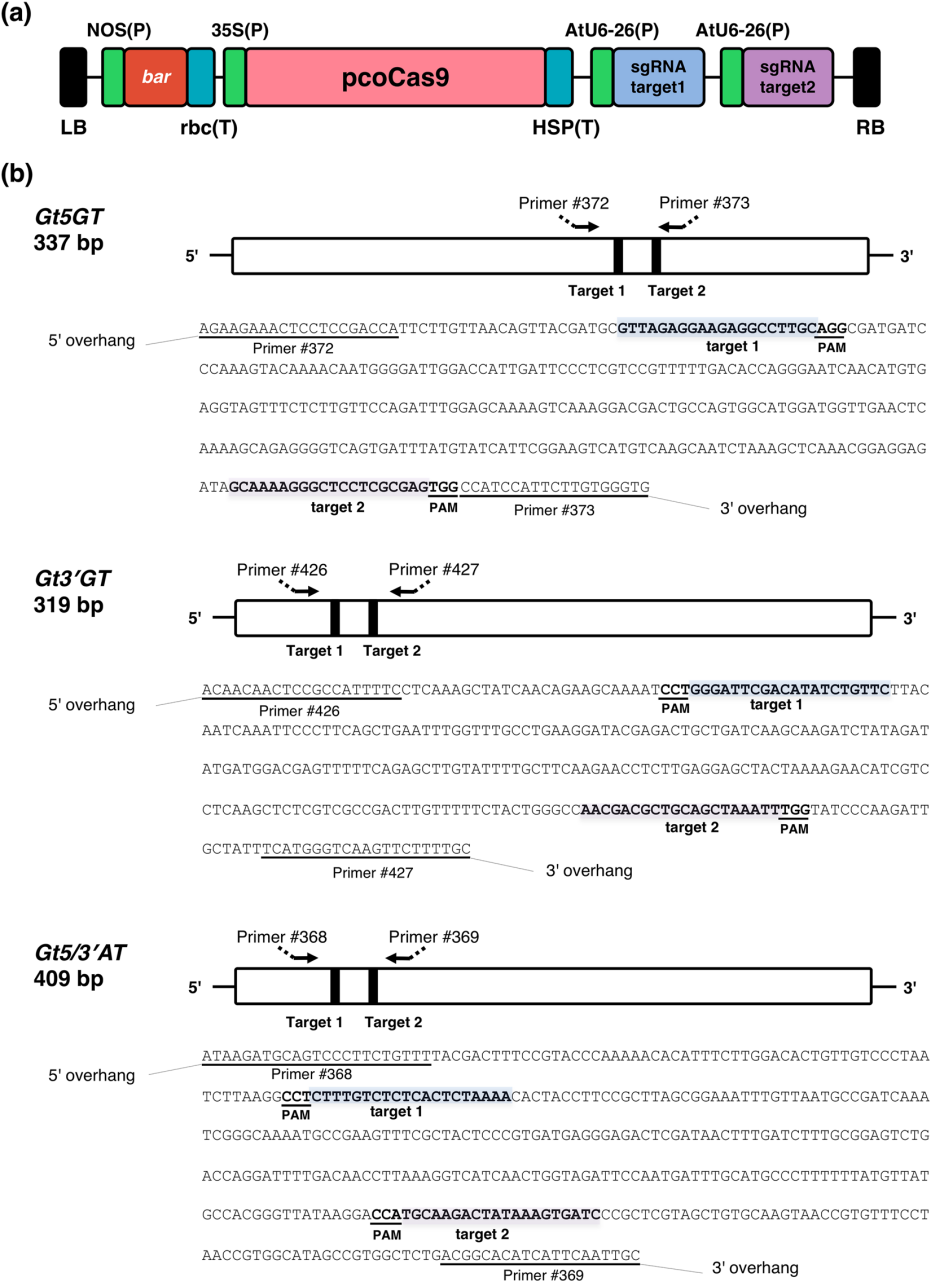
Schematic diagram of the CRISPR/Cas9 construct and the target sites of three anthocyanin modification genes. (**a**) Schematic diagram of the T-DNA region of the CRISPR/Cas9 binary vector carrying pcoCas9, which is a codon-optimized Cas9 for plants. Two sgRNA expression cassettes are tandemly aligned. (**b**) Schematic diagrams of the *Gt5GT*, *Gt3*′*GT*, and *Gt5/3*′*AT* genes and their sequences, including the sequences of the amplified regions. Shaded boxes represent targets 1 and 2, whereas PAM refers to the protospacer adjacent motif (NGG). The primers used for the PCR amplification or direct sequencing are presented.

Adventitious shoots were selected on medium containing 0.5 or 0.75 mg l^−1^ bialaphos. A total of 35, 41, and 37 bialaphos-resistant putative transgenic lines were obtained for the transformation experiments involving the *Gt5GT*, *Gt3*′*GT*, and *Gt5/3*′*AT* constructs, respectively, of which 23, 36, and 31 lines, respectively, were confirmed to be transgenic by the PCR amplification of part of the pcoCas9 sequence using crude DNA samples. The genome-edited shoots were then screened by a direct Sanger sequence analysis of the PCR products amplified by the primer sets presented in Fig. 2b. Several clones of the PCR products from potential genome-edited shoots were subjected to further subcloning, and the sequences, including the target sites, were determined. Finally, we obtained two, two, and five lines that may have biallelic mutations at the *Gt5GT*, *Gt3*′*GT*, and *Gt5/3*′*AT* target sites, respectively. For each transformed construct, two genome-edited knockout lines were selected and grown in a closed greenhouse until flowering.

### Next-generation sequencing analysis of genome-edited gentian plants

The results of the amplicon sequence analyses of all target sites in the transformants are summarized in Table 1. We excluded non-specific or low-copy fragments that were thought to be derived from PCR and/or sequence errors, and determined the number of major fragments of each allele. The number of the major *Gt5GT*, *Gt3*′*GT*, and *Gt5/3*′*AT* genomic DNA amplicons in the WT plants represented 68.8% (24,402/35,448), 72.7% (21,762/29,916), and 75.8% (33,661/44,435) of the total number of fragments, respectively. Analyses of SNP sequences revealed the gentian genome includes two alleles for *Gt5GT* and *Gt5/3*′*AT*, but four alleles for *Gt3*′*GT*. The counted read numbers among individual gene alleles of RNA were almost equal (less than 2.5-fold) in WT plants, except for *Gt3*′*GT* allele 4, which was counted approximately 5- to 10-fold higher than that of alleles 1–3. Amplicon sequence analyses also indicated that each transformant contained several characteristic mutations, such as deletions of 1–30 bases or more than 200 bases between the target 1 and target 2 sequences, depending on the transgenic lines and target genes (Table 1). All *Gt5GT* and *Gt5/3*′*AT* transformants were confirmed to have heterozygous biallelic mutations. Interestingly, the inversion of a 202-base sequence and a 27-base deletion were also confirmed in one allele of *Gt5/3*′*AT* #50. Additionally, we were unable to distinguish between alleles 2, 3, and 4 in *Gt3*′*GT* #11 because of a lack of SNPs due to a 221-bp deletion. Regarding *Gt3*′*GT* #35, some alleles were not detected, probably because of one or more large deletions or base changes to primer sites. However, for both *Gt3*′*GT* lines, the remaining amplified alleles were affected by mutations that caused frameshifts. Although the ratio of fragment counts for each allele differed between the genomic DNA and transcripts, we detected a consistent mutation pattern between the genomic DNA and transcripts in all transgenic lines. Therefore, these transgenic lines were confirmed as gene knockout lines, and they were analyzed further.

**Table 1:** NGS analysis of PCR amplicons of DNA and RNA of genome-edited lines.

Target gene	Line	Allele	No. of counts (DNA)	No. of counts (RNA)	Target 1		Target 2	
					Sequence	In/Del	Sequence	In/Del
*Gt5GT*	WT	1	12,79–8	50,836	**GTTAGAGGAAGAGGCCTTGC** **AGG**	0	**GCAAAAGGGCTCCTCGCGAG** **TGG**	0
		2	11,604	26,024	**GTTAGAGGAAGAGGCCTTGC** **AGG**	0	**GCAAAAGGGCTCCTCGCGAG** **TGG**	0
	#47	1	9,611	6,443	**GTTAGAGGAAGAGGCCTTGC** **AGG**	0	**GCAAAAGGGCT-----CGAG** **TGG**	−5
		2	9,605	36,768	**GTTAGAGGAAGAGGCCTTGC** **AGG**	0	**GCAAAAGGG---------AG** **TGG**	−9
	#106	1	18,816	29,042	**CGTTAGAGGAAGAGGCC-TGC** **AGG**	−1	**GCAAAAGGG-------CGAG** **TGG**	−7
		2	30,010	261,382	**CGTTAGAGGAAGAGGC----------------||----------------GAG** **TGG**			−256
*Gt3*′*GT*	WT	1	5,666	4,852	**CCT** **GGGATTCGACATATCTGTTC**	0	**AACGACGCTGCAGCTAAATT** **TGG**	0
		2	5,419	2,055	**CCT** **GGGATTCGACATATCTGTTC**	0	**AACGACGCTGCAGCTAAATT** **TGG**	0
		3	5,825	2,018	**CCT** **GGGATTCGACATATCTGTTC**	0	**AACGACGCTGCAGCTAAATT** **TGG**	0
		4	4,852	22,326	**CCT** **GGGATTCGACATATCTGTTC**	0	**AACGACGCTGCAGCTAAATT** **TGG**	0
	#11^*^	1	20,194	35,543	**CCT** **GGG------CATATCTGTTC**	−6	**AACGACGCTGCAGCT-AATT** **TGG**	−1
		2	34,884	461,515	**CCT** **GGG---------------------||--------------------AATT** **TGG**			−221
		3						
		4						
	#35	1	7,186	3,004	**CCT** **-------GACATATCTGTTC**	−7	**AACGACGCTGCAGCTAAATT** **TGG**	0
		2	7,223	33,855	**--||--ATTCGACATATCTGTTC**	−10	**AACGACGCTGCAGCTAAATT** **TGG**	0
		3	—	—	not amplified	—	not amplified	—
		4	—	—	not amplified	—	not amplified	—
*Gt5/3*′*AT*	WT	1	15,403	12,453	**CCT** **CTTTGTCTCTCACTCTAAAA**	0	**CCA** **TGCAAGACTATAAAGTGATC**	0
		2	18,258	20,721	**CCT** **CTTTGTCTCTCACTCTAAAA**	0	**CCA** **TGCAAGACTATAAAGTGATC**	0
	#50	1	16,376	15,213	**CCT** **CTTTGTCTCTCACTCTAAAA**	0	**------||-------AAGTGATC**	−30
		2	19,913	11,267	**CCT** **CTT-----||------ [202 base reversed] AGACTATAAAGTGATC**			−27
	#60	1	7,872	1,272	**CCT** **CTTTGTCTCTCACTCTAAAA**	0	**CCA** **TGC----CTATAAAGTGATC**	−4
		2	6,874	22,102	**CCT** **C---GTCTCTCACTCTAAAA**	−3	**CCA** **TGC–ACTATAAAGTGATC**	−3

Double underlines show PAM sequences.

*Allele 2, 3 and 4 are indistinguishable from each other due to large deletion.

### Petal phenotypes of the genome-edited gentian plants

**Figure 3:**
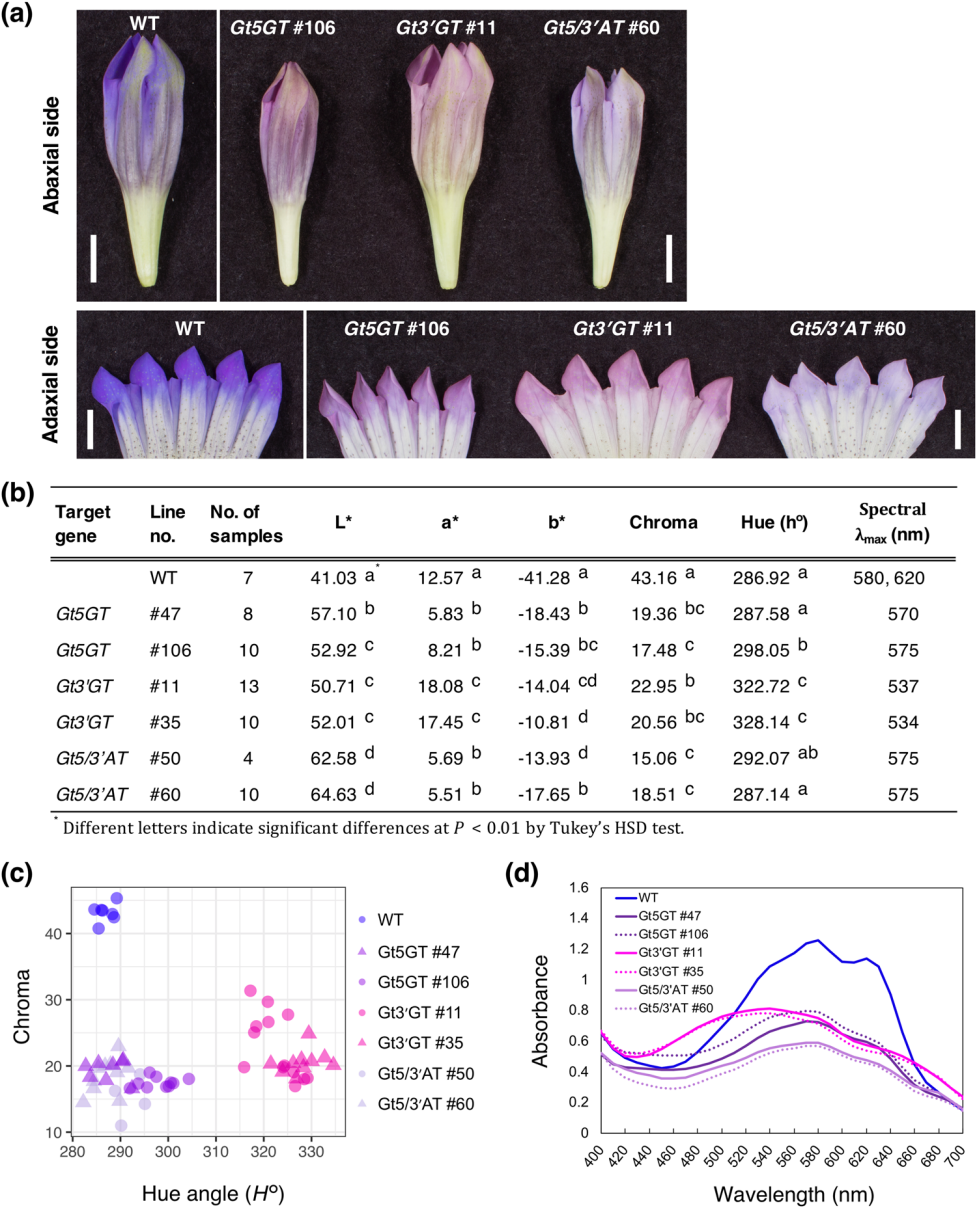
Characteristics of flower colors in genome-edited gentian plants. (**a**) Abaxial and adaxial sides of the flower petals of the wild-type (WT) plants and the *Gt5GT*, *Gt3*′*GT*, and *Gt5/3*′*AT* lines. Bar = 1 cm. (**b**) The L* a* b* color values and visible spectral data measured spectrophotometrically of the surface of fresh petals are provided. Different letters indicate significant differences at *P* < 0.01 according to Tukey’s HDS test. (**c**) Color distribution visualized by a 2-dimensional scatter plot based on L* and chroma values. (**d**) Absorption spectra of the surface of fresh petals in WT and genome-edited knockout lines measured spectrophotometrically. The spectra are based on the mean data for the petals described in (**b**).

At approximately 4 months after plants were first acclimated, we detected changes to the flower color phenotypes in the transformants in a closed greenhouse. Figure 3a presents the abaxial and adaxial sides of flower petals of representative transformants. Specifically, the *Gt5GT* #106, *Gt3*′*GT* #11, and *Gt5/3*′*AT* #60 lines produced pale red violet, dull pink, and pale mauve flowers, respectively. The colorimetric values of the adaxial side of the flower limb area for the *Gt5GT* (#47 and #106), *Gt3*′*GT* (#11 and #35), and *Gt5/3*′*AT* (#50 and #60) transformants are summarized in Fig. 3b. The L*, a*, b*, and C* values for all transformants were significantly higher and/or lower than the corresponding values for the WT plants (Fig. 3b). The hue angles of *Gt3*′*GT* (#11 and #35) plants, which produced flowers with dull pink petals, exceeded 300°. The C* values and hue angles are presented in a scatter plot to visualize the color distribution (Fig. 3c). Although the two-dimensional coordinated positions of the *Gt5GT* and *Gt5/3*′*AT* lines overlapped (Fig. 3c), the L* values of the *Gt5/3*′*AT* lines were significantly higher than those of the *Gt5GT* lines (Fig. 3b). Moreover, the hue angles were significantly greater in the *Gt3*′*GT* lines than in the WT plants and in the other transformants. Furthermore, the C* value was significantly lower in all transformants than in the WT plants. Thus, the color distribution clearly indicated that all transformants differed from the WT plants. The absorption spectra of the transformants are provided in Fig. 3d. The absorbance values were high for the WT plants, with two peaks (at 580 and 620 nm). In contrast, all transformants had absorbance values less than 1.0, with a slight decrease at 600 nm. The absorbance of the *Gt3*′*GT* lines shifted more intensely at shorter wavelengths than the *Gt5GT* and *Gt5/3*′*AT* lines.

### Analysis of anthocyanins in genome-edited gentian plants

**Figure 4:**
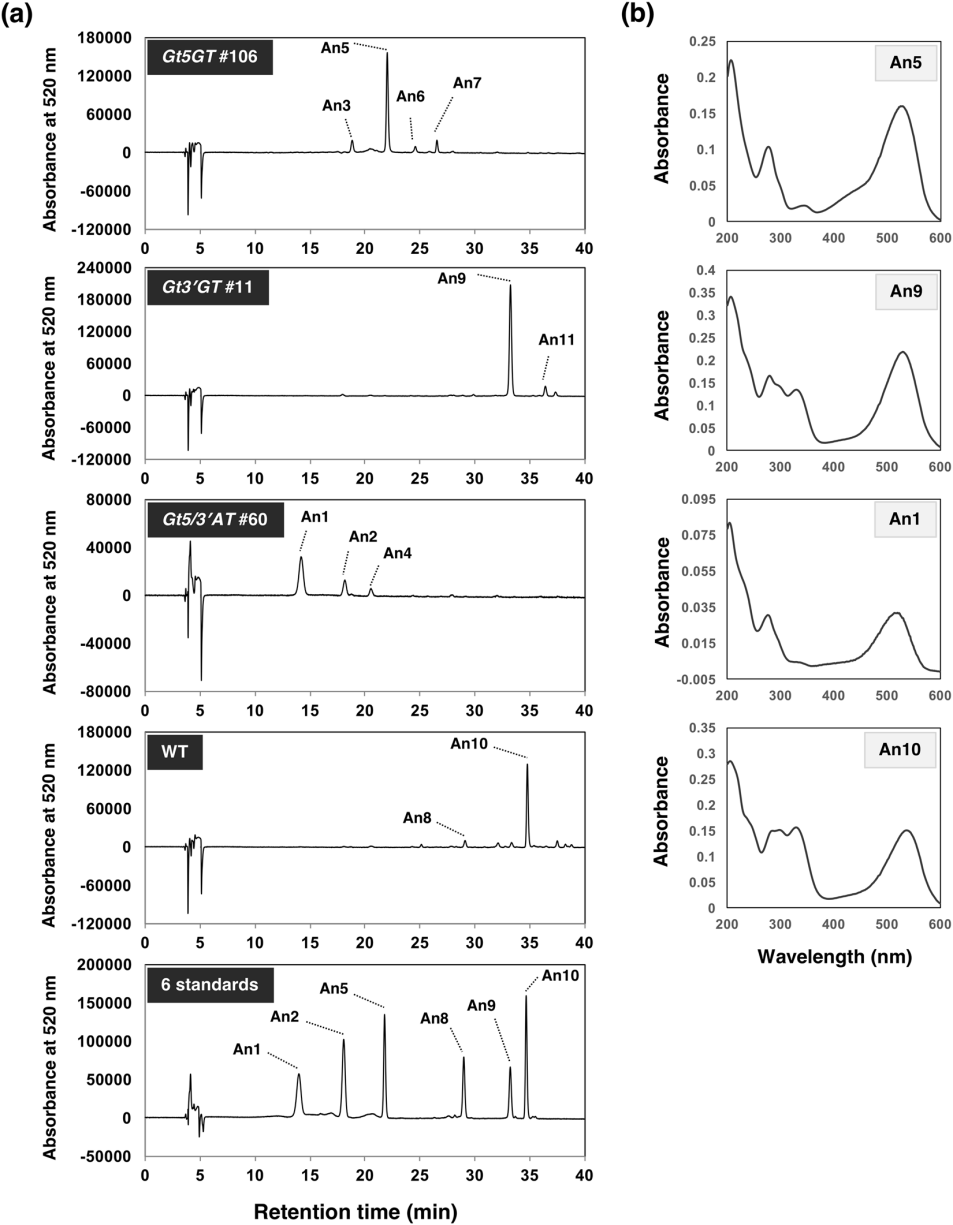
Anthocyanin profiles of the flower petals of the genome-edited gentian plants. (**a**) HPLC chromatograms (at 520 nm) of the anthocyanins extracted from the petals of the *Gt5GT* #106, *Gt3*′*GT* #11, and *Gt5/3*′*AT* #60 lines. An1, delphinidin 3,5,3′-triglucoside; An2, delphinidin 3,5-diglucoside; An5, delphinidin 3-glucoside; An8, delphinidin 3-glucosyl-5-caffeoyl-glucosyl-3′-glucoside; An9, delphinidin 3-glucosyl-5-caffeoyl-glucoside; An10, delphinidin 3-glucosyl-5-caffeoyl-glucosyl-3′-caffeoyl-glucoside (gentiodelphin); An3, 4, 6, 7, and 11 are unknown anthocyanins. (**b**) Spectra of the major peaks in *Gt5GT*#106 (An5), *Gt3*′*GT*#11 (An9), *Gt5/3*′*AT* #60 (An1), and WT (An10).

The petal extracts of the transformants and WT control were separated by HPLC (at 520 nm). The chromatograms and major peaks of *Gt5GT* #106, *Gt3*′*GT* #11, *Gt5/3*′*AT* #60, and WT plants (i.e., the same lines as in Fig. 3a) are presented in Fig. 4a,b. Eleven peaks were detected in the samples. Minor peaks (peak area <100,000) were excluded from analyses. We designated the peaks as An1 to An11 based on the retention times. A co-chromatography and analysis of absorbance spectra indicated peaks An1, An2, An5, An8, An9, and An10 corresponded to six standards. Anthocyanin compositions calculated based on the peak area at 520 nm are summarized in Table 2. In WT plants, one major peak (An10) corresponding to gentiodelphin accounted for 91% of the total peak area, whereas gentiodelphin was undetectable in all transformants. In the *Gt5GT*, *Gt3*′*GT*, and *Gt5/3*′*AT* lines, the major peaks were An5, An9, and An1, which corresponded to delphinidin 3-glucoside (61% or 74%), delphinidin 3-glucosyl-5-caffeoyl-glucoside (90% or 100%), and delphinidin 3,5,3′-triglucosides (57% or 75%), respectively. The presence of minor peaks implied a small amount of unidentified anthocyanins accumulated in the transformants (Fig. 4a and Table 2). Similar results were obtained for other genome-edited lines (Supplementary Fig. S1). The anthocyanin concentrations in flower petal extracts were calculated and expressed as delphinidin equivalents (Supplementary Fig. S2). *Gt5GT*-edited lines showed significant increase in anthocyanin concentrations compared with WT and other genome-edited lines.

**Table 2:** Anthocyanin-content ratio calculated from peak area of HPLC at 520 nm (%).

plant		An1	An2	An3	An4	An5	An6	An7	An8	An9	An10	An11
		DEL 3G-5G-3′G	DEL 3G-5G			DEL 3 G			DEL 3G-5CafG-3′G	DEL 3G-5CafG	Gentiodelphin	
WT							1		8		91	
*Gt5GT*	#47			29		61		10				
	#106			11		74	8	7				
*Gt3*′*GT*	#11									90		10
	#35									100		
*Gt5/3*′*AT*	#50	57	35		8							
	#60	75	25									

## Discussion

In this study, we used the CRISPR/Cas9 system to edit three anthocyanin modification genes (*Gt5GT*, *Gt3*′*GT*, and *Gt5/3*′*AT*) in the Japanese gentian genome. In preliminary experiments, we obtained genome-edited gentian plants by applying one sgRNA targeting one site. However, because the frequency of the genome editing greatly varied depending on the genes and target sites, we decided to use two sgRNAs to target two sites on a single gene (Fig. 2b). The use of multiple sgRNAs reportedly increases the efficiency of Cas9-mediated genome editing by increasing the mutation rate of the target genes^20–22^. It is also expected to introduce large fragment deletions between the two target sites. In the current study, we obtained one line (*Gt3*′*GT* #11) with a large sequence deletion (Table 1). Additionally, the biallelic mutation efficiencies based on a Sanger sequence analysis were 5.7% (2/35 lines), 4.9% (2/41 lines), and 8.6% (5/58 lines) for the *Gt5GT*, *Gt3*′*GT*, and *Gt5/3*′*AT* transformants, respectively (data not shown). These efficiencies are much lower than the reported efficiencies for other plants, such as torenia (80%)^18^, morning glory (75%)^17^, and rice (67.6%)^23^. The reason for this difference remains unclear, but one possibility is that the CaMV 35 S promoter-mediated expression of *SpCas9* is considerably silenced in gentian^24^. Another possibility is that the high heterogeneity of the large gentian genome (approximately 5 Gb, 2n = 2 × = 26) contributes to the observed inefficiencies. Our NGS analysis of DNA and RNA revealed that the number of major allele fragments represented 66.9%–95.4% of the total number of fragments (Supplementary Table S2). The minor fragments contained SNPs and/or In/dels around the target sequence, but these variations were likely derived from PCR or NGS errors. Alleles 3 and 4 in *Gt3*′*GT*-edited line #35 were not detected in our NGS analysis, suggesting the mutations were located outside the sequenced region amplified with primers #426 and #427. An analysis of RNA amplicons indicated that the number of non-mutated WT allele fragments accounted for less than 0.05% of the total number of fragments. This result may have been due to technical errors during the NGS analysis or because of the extremely low chimerism of the remaining unedited cells. However, the expression of a few non-edited sequences in gentian flowers is unlikely to affect the absorbance of the petal surface evaluated with a spectrophotometer or the peak area values determined by HPLC. Thus, we concluded that *Gt5GT*-, *Gt3*′*GT*-, and *Gt5/3*′*AT*-edited lines can be used to evaluate flower coloration during an *in vivo* analysis. To increase the efficiency of gentian genome editing, we are now assessing the effects of several modifications to the CRISPR/Cas9 system, including to the promoters, the sgRNA scaffold structure, and the selection conditions during the *A*. *tumefaciens*-mediated transformation step.

Knocking out *Gt3*′*GT* induced the accumulation of delphinidin 3G-5CafG as the major anthocyanin in the petal tissue of lines #11 (90%) and #35 (100%) (Table 2). The *Gt3*′*GT-*edited knockout lines did not accumulate delphinidin 3G-5G in petals, suggesting the glucose moiety at the 5-position was efficiently acylated by Gt5/3′AT. In contrast, the *Gt5/3*′*AT*-edited knockout lines accumulated not only delphinidin 3G-5G-3′G, but also some delphinidin 3G-5G (Fig. 4, Table 2), likely because of the incomplete Gt3′GT-catalyzed modification of delphinidin 3G-5G. As presented in Fig. 1, the modification pathway, which had been biochemically verified in a previous study^15^, branches into two pathways so that delphinidin 3G-5G may be glucosylated by Gt3′GT (pathway 1) or acylated by Gt5/3′AT (pathway 2). Additionally, the residual delphinidin 3G-5G may be used as a substrate for pathway 2. Thus, our analyses of the *Gt3*′*GT*- and *Gt5/3*′*AT*-knockout gentian plants indicate that both pathways contribute to gentiodelphin biosynthesis in gentian petals. Unidentified anthocyanins also accumulated in *Gt5GT*-edited knockout lines. It is probable that any of An3, An6, and An7 corresponds to delphinidin 3,3′-diglucoside or delphinidin 3-glucosyl-3′-caffeoyl-glucoside, because the recombinant Gt3′GT protein has slight glucosylation activity against delphinidin 3-glucoside^15^. It is also notable that *Gt5/3*′*AT* lines accumulated no acylated anthocyanins and *5GT* lines accumulated anthocyanins where there are no side groups at the 5 position as major pigments, suggesting that CRISPR/Cas9 system could produce knockout lines for the specific glucosyl and acyl transferase genes.

The absorption spectrum of the surface of the fresh petals of WT plants revealed the highest absorbance occurred at 580 nm, with an additional peak at 620 nm (Fig. 3d). The characteristic peaks detected for the WT gentian petals were also observed for the petals of other plant species with blue flowers, including *Commelina communis* (metal complex pigment), *Platycodon grandiflorus* (polyacylated anthocyanin), and *Felicia amelloides* (intermolecular co-pigmentation with flavone *C*-glycosides) (Supplementary Fig. S3). However, the absorption spectra of the *Gt5GT-*, *Gt3*′*GT-*, and Gt*5/3*′*AT*-edited knockout lines were lower than that of the WT control, with a slight decrease at 600 nm. The a*, b*, L*, and C* values of the genome-edited knockout lines accumulating intermediate delphinidin derivatives varied from the corresponding values of the WT plants (Fig. 3b). Specifically, the b* value for blue–yellow color components (blue in the negative direction) considerably increased in all transformants.

Flower color is determined by not only the anthocyanin kinds but also the anthocyanin amounts in petals. Our result indicated that *Gt5GT*-edited lines showed about 2-fold increase in anthocyanin concentrations compared with WT and other lines (Supplementary Fig. S2). This may also contribute to the observed change of flower color in *Gt5GT* lines. Although the reason remains unknown, anthocyanin transport activity and/or anthocyanin stability in vacuole may be different among anthocyanin molecules. Further analysis is needed to elucidate the effects of anthocyanin modifications on flower color pigmentation in gentian. However, our results clearly confirmed the actual gentiodelphin biosynthetic pathway and its contribution to blue color development previously only reported from *in vitro* chemical analysis.

In summary, we confirmed the importance of gentiodelphin for the blue coloration of gentian flowers by analyzing genome-edited lines in which specific anthocyanin modification genes were knocked out by the CRISPR/Cas9 system. Our data provide novel insights regarding the gentiodelphin biosynthetic pathway and verify the co-pigmentation bluing effects on flower petals. We are now developing new CRISPR/Cas9 vectors and optimizing the method for selecting genome-edited knockout lines. We are also generating various genome-edited knockout lines suitable for horticultural, physiological, and pathological studies. We believe the CRISPR/Cas9 system will accelerate future gentian studies.

## Materials and Methods

### Plant materials and transformation

The blue-flowered gentian cv. Albireo (*Gentiana triflora* × *Gentiana scabra*) plants that were transformed in this study were maintained in an *in vitro* culture. Half-strength Murashige and Skoog (MS) medium containing 3% (w/v) sucrose and 0.2% (w/v) gellan gum was used as the basal culture medium. The growth conditions in an incubation room were as follows: 22 °C under a 16-h light/8-h dark photoperiod, with light supplied by cool white fluorescent lamps. Plants were transformed according to a modified version of a previously described *Agrobacterium tumefaciens*-mediated procedure^24^. Briefly, leaf sections were excised and co-cultivated with *A*. *tumefaciens* cells harboring a CRISPR/Cas9 vector (Fig. 2a) on half-strength MS medium supplemented with 100 μM acetosyringone. After a 5-day co-cultivation, the leaf sections were transferred to selection medium containing 0.5 or 0.75 mg l^−1^ bialaphos (Meiji-Seika Co. Ltd., Tokyo, Japan), 500 mg l^−1^ cefotaxime (Claforan; Aventis Pharma, France), 10 mg l^−1^ thidiazuron [N-phenyl-N′-(1,2,3-thiadiazol-5-yl) urea], and 1 mg l^−1^ 1-naphthaleneacetic acid. Adventitious shoots were induced from calli dedifferentiated from the edge of leaves on selection medium and transferred to half-strength MS medium solidified with 0.25% (w/v) gellan gum and supplemented with 3% (w/v) sucrose, 500 mg l^−1^ cefotaxime, and 0.75 mg l^−1^ bialaphos for rooting. The production of transgenic material was first confirmed by PCR, which was completed with crude extracts from shoots, MightyAmp DNA polymerase (TaKaRa, Shiga, Japan), and primers #61_Cas9U1898 and #62_Cas9L2260 specific for pcoCas9 (Supplementary Table S1). The regenerated plants were acclimated as previously described^25^.

### Construction of binary vectors for editing the gentian genome

Binary CRISPR/Cas9 vectors (pSbar-pcoCas9_AtU6-26p-target-gRNA_AtU6-26p-target-gRNA) (Fig. 2) targeting gentian *Gt5GT*, *Gt3*′*GT*, and *Gt5/3*′*AT* genes were constructed for a subsequent plant transformation. A basic vector, pSbar-pcoCas9, was first constructed by replacing the *uidA* (*gus*) gene of pSKAN221-HT^26^ with the plant codon-optimized *SpCas9* (pcoCas9)^27^. Two single-guide RNA (sgRNA) expression cassettes targeting the gentian *Gt5GT*, *Gt3*′*GT*, and *Gt5/3*′*AT* genes were introduced into pSbar-pcoCas9 in tandem via a restriction enzyme digestion and ligation. Target sites for each gene are presented in Fig. 2b. Finally, three binary vectors, pSbar-pcoCas9-AtU6-26p-Gt5GTtarget1gRNA-AtU6-26p-t5GTtarget2gRNA, pSbar-pcoCas9-AtU6-26p-Gt5/3′ATtarget1gRNA-AtU6-26p-Gt5/3′ATtarget2gRNA, and pSbar-pcoCas9-AtU6-26p-Gt3′GTtarget1gRNA-AtU6-26p-Gt3′GTtarget2gRNA, were inserted into *A*. *tumefaciens* strain EHA101 cells by electroporation.

### Measurement of gentian petal colors

To evaluate the flower color phenotypes of the genome-edited knockout lines and the untransformed wild-type (WT) plants, the adaxial surface of freshly collected petals was analyzed spectrophotometrically. For each line, the colorimetric values (L*, a*, and b*) and the absorption spectra of the adaxial surface of the limb area of fresh petals were measured with the CM-3600A spectrophotometer (Konica Minolta, Tokyo, Japan). The chroma C* value and the hue angle were also calculated. Tukey’s HSD test was performed to test differences among sample means for significance.

### Anthocyanin analysis

The anthocyanin compositions of petal extracts were analyzed by HPLC. Fresh petals were collected from greenhouse-grown plants and stored at −80 °C until used. The petals were soaked in 80% methanol containing 0.1% (v/v) trifluoroacetic acid to extract the anthocyanins, which were then separated with an HPLC–photodiode array detector system [PU-4180 PUMP, MD-4015 photodiode array detector, and ChromNAV (version 2.02) software; JASCO, Tokyo, Japan] equipped with a COSMOSIL PBr column (4.6 mm internal diameter × 250 mm; Nacalai Tesque, Kyoto, Japan). We used a linear gradient elution (2 ml min^−1^) of 10%–80% acetonitrile in 1% aqueous phosphoric acid over 50 min.

Total anthocyanin concentrations were estimated based on the molar absorptivity of delphinidin (ε mol = 27,940 at 520 nm, evaluated in 80% MeOH containing 0.1% TFA). Delphinidin chloride, delphinidin 3-*O*-glucoside and delphinidin 3,5-*O*-diglucoside were purchased from Funakoshi Co. (Tokyo, Japan). Additionally, delphinidin 3-*O*-*β*-d-glucoside 5-*O*-(6-*O*-caffeoyl-*β*-d-glucoside) 3′-*O*-*β*-d-glucoside, delphinidin 3-*O*-*β*-d-glucoside 5-*O*-(6-*O*-caffeoyl-*β*-d-glucoside), delphinidin 3,5,3′-*O*-triglucoside (kindly provided by Drs. Tanaka and Nakamura, Suntory Ltd.), and gentiodelphin [delphinidin 3-*O*-*β*-d-glucoside 5-*O*-(6-*O*-caffeoyl-*β*-d-glucoside) 3′-*O*-(6-*O*-caffeoyl-*β*-d-glucoside)] were used as authentic standards.

### Sanger sequencing analysis

The leaves of transgenic plant lines were analyzed by PCR with MightyAmp DNA polymerase according to the manufacturer’s instructions. Details regarding the primers designed for the *Gt5GT*, *Gt3*′*GT*, and *Gt5/3*′*AT* genes are provided in Supplementary Table S1. The PCR products were purified and sequenced with the BigDye Terminator (version 1.1) Cycle Sequencing Kit and the ABI PRISM 3130xl or 3500 Genetic Analyzers (Applied Biosystems, Foster City, CA, USA).

### Next-generation sequencing analysis

The genomic DNA and transcripts in the petals of the genome-edited lines and the WT control underwent an Illumina NGS analysis. Genomic DNA was isolated from the leaves of transgenic and WT gentian plants with the GeneElute Genome DNA Isolation system (Sigma-Aldrich, St Louis, MO, USA). Total RNA was isolated from the petals of transgenic gentian plants with the RNeasy Plant Mini kit (Qiagen, GmbH, Hilden, Germany) and treated with DNase I (TaKaRa) according to the manufacturer’s instructions. The extracted RNA samples were reverse transcribed with PrimeScript II (TaKaRa). The subsequent library preparation, PCR amplification, amplification check, and library quantification check were performed as previously described^18^. The primer positions and target sites are indicated in Fig. 2b. Additional details regarding the primers (e.g., index) are listed in Supplementary Table S1. A bioinformatics analysis was performed as previously described^18^. Briefly, the resulting raw sequence reads were pre-processed with the FASTX toolkit, and the read pairs were merged into a single contiguous sequence (fragment) with a fastq-join script^28^. The unique fragments were then counted.

## Supplementary information

Supplementary information
