# Supplementary material for: Effects of knocking out three anthocyanin modification genes on the blue pigmentation of gentian flowers
Source: Sci Rep. 2019 Nov 1;9:15831. doi: 10.1038/s41598-019-51808-3 (PMC6825144; doi:10.1038/s41598-019-51808-3)
Supplement: Supplementary file 1 — Supplementary information [file 41598_2019_51808_MOESM1_ESM.pdf]

Supplementary information

Effects of knocking out three anthocyanin modification genes on the blue pigmentation of gentian flowers

Keisuke Tasaki[1,2], Atsumi Higuchi[1], Aiko Watanabe[1], Nobuhiro Sasaki[1,3], Masahiro Nishihara[1*]

[1] Iwate Biotechnology Research Center, 22-174-4 Narita, Kitakami, Iwate 024-0003, Japan

[2] Present address: Tokyo University of Agriculture, Atsugi, Kanagawa 243-0034, Japan

[3] Present address: Toyo University, 1-1-1 Izumino, Itakura-machi, Ora-gun, Gunma 374-0193, Japan

Supplementary Table S1

Supplementary Table S2

Supplementary Figure S1

Supplementary Figure S2

Supplementary Figure S3

Supplementary Table S1. Primers used in this study

Primer Name	Sequence	Index	Note
**Direct sequnce primer sets**			
**#61_Cas9U1898**	AAGAAAGGCTCAAGACCTACGCTCAT	-	Forward primer
**#62_Cas9L2260**	TCGATCACGATGTTCTCAGGCTTATG	-	Reverse primer
**Gt5GT_U667**	AAAGAGCACCTGGAAGCACTTG	-	Forward primer / Used for sequencing reaction
**Gt5GT_L1147**	GCTCAGTTTCAGGTGCTAAAGC	-	Reverse primer
**#144_Gt5/3'AT-U6**	GCAAATCCAAATGGTGAAGG	-	Forward primer / Used for sequencing reaction
**#145_Gt5/3'AT-L573**	ATAGGCCCAAGCATTGATGA	-	Reverse primer
**#149_Gt3'GT-U2**	TGGATCAGCTTCACGTTTTC	-	Forward primer
**#150_Gt3'GT-L514**	GCACCACAAAAGGGTCAGAA	-	Reverse primer / Used for sequencing reaction
**Amplicon sequence primers**			
**1st PCR primer sets**			
**#368_Alb.Gt5/3'ATtar1,3_D50X**	ACACTCTTTCCCTACACGACGCTCTTCCGATCtATAAGATGCAGTCCCTTCTGTTT	-	Forward primer
**#369_Alb.Gt5/3'ATtar1,3_D70X**	TGACTGGAGTTCAGACGTGTGCTCTTCCGATCtGCAATTGAATGATGTGCCGT	-	Reverse primer
**#372_Alb.Gt5GTtar1,2_D50X**	ACACTCTTTCCCTACACGACGCTCTTCCGATCtAGAAGAAACTCCTCCGACCA	-	Forward primer
**#373_Alb.Gt5GTtar1,2_D70X**	TGACTGGAGTTCAGACGTGTGCTCTTCCGATCtCACCCACAAGAATGGATGG	-	Reverse primer
**#426_Alb.Gt3'GTtar1,3_D50X**	ACACTCTTTCCCTACACGACGCTCTTCCGATCtACAACAACTCCGCCATTTTC	-	Forward primer
**#427_Alb.Gt3'GTtar1,3_D70X**	TGACTGGAGTTCAGACGTGTGCTCTTCCGATCtGCAAAAGAACTTGACCCATGA	-	Reverse primer
**2nd PCR forward primers**			
**D501**	AATGATACGGCGACCACCGAGATCTACACTATAGCCTACACTCTTTCCCTACACGACG	TATAGCCT	
**D502**	AATGATACGGCGACCACCGAGATCTACACATAGAGGCACACTCTTTCCCTACACGACG	ATAGAGGC	
**D503**	AATGATACGGCGACCACCGAGATCTACACCCTATCCTACACTCTTTCCCTACACGACG	CCTATCCT	
**D504**	AATGATACGGCGACCACCGAGATCTACACGGCTCTGAACACTCTTTCCCTACACGACG	GGCTCTGA	
**D505**	AATGATACGGCGACCACCGAGATCTACACAGGCGAAGACACTCTTTCCCTACACGACG	AGGCGAAG	
**D506**	AATGATACGGCGACCACCGAGATCTACACTAATCTTAACACTCTTTCCCTACACGACG	TAATCTTA	
**D507**	AATGATACGGCGACCACCGAGATCTACACCAGGACGTACACTCTTTCCCTACACGACG	CAGGACGT	
**D508**	AATGATACGGCGACCACCGAGATCTACACGTACTGACACACTCTTTCCCTACACGACG	GTACTGAC	
**2nd PCR reverse primers**			
**D701**	CAAGCAGAAGACGGCATACGAGATCGAGTAATGTGACTGGAGTTCAGACGTGT	ATTACTCG	
**D702**	CAAGCAGAAGACGGCATACGAGATTCTCCGGAGTGACTGGAGTTCAGACGTGT	TCCGGAGA	
**D703**	CAAGCAGAAGACGGCATACGAGATAATGAGCGGTGACTGGAGTTCAGACGTGT	CGCTCATT	
**D705**	CAAGCAGAAGACGGCATACGAGATTTCTGAATGTGACTGGAGTTCAGACGTGT	ATTCAGAA	
**D706**	CAAGCAGAAGACGGCATACGAGATACGAATTCGTGACTGGAGTTCAGACGTGT	GAATTCGT	
**D707**	CAAGCAGAAGACGGCATACGAGATAGCTTCAGGTGACTGGAGTTCAGACGTGT	CTGAAGCT	
**D708**	CAAGCAGAAGACGGCATACGAGATGCGCATTAGTGACTGGAGTTCAGACGTGT	TAATGCGC	

Supplementary Table S2. Summary of NGS amplicon analyses of target sites

Line	Nucleotide	Number of total fragment reads	Number of Fragment patterns	Allele 1	Allele 2	Allele 3	Allele 4	Total alleles	Total alleles / total fragments	Non-mutated allele 1	Non-mutated allele 2	Non-mutated allele 3	Non-mutated allele 4	Non-mutated alleles / total fragments
Gt5GT#47	**DNA**	25,251	1,831	9,611	9,605	-	-	19,216	76.1%	17	0	-	-	0.07%
Gt5GT#106	**DNA**	59,169	1,836	18,816	30,010	-	-	48,826	82.5%	0	0	-	-	0.00%
Gt3'GT#11	**DNA**	74,539	2,210	20,194	35,543	-	-	55,737	74.8%	0	0	0	0	0.00%
Gt3'GT#35	**DNA**	21,531	1,288	7,186	7,223	-	-	14,409	66.9%	0	0	0	0	0.00%
Gt5/3'AT#50	**DNA**	46,338	2,243	16,376	15,213	-	-	31,589	68.2%	0	0	-	-	0.00%
Gt5/3'AT#60	**DNA**	19,008	2,552	7,872	6,874	-	-	14,746	77.6%	2	1	-	-	0.02%
Gt5GT#47	**RNA**	49,649	1,931	6,443	36,768	-	-	43,211	87.0%	0	0	-	-	0.00%
Gt5GT#106	**RNA**	305,289	2,281	29,042	261,382	-	-	290,424	95.1%	26	0	-	-	0.01%
Gt3'GT#11	**RNA**	520,911	3,405	35,543	461,515	-	-	497,058	95.4%	63	0	54	0	0.02%
Gt3'GT#35	**RNA**	41,466	2,063	3,004	33,855	-	-	36,859	88.9%	0	1	0	10	0.03%
Gt5/3'AT#50	**RNA**	33,394	3,212	19,913	11,267	-	-	31,180	93.4%	11	5	-	-	0.05%
Gt5/3'AT#60	**RNA**	34,795	2,046	1,272	22,102	-	-	23,374	67.2%	0	0	-	-	0.00%
**WT *Gt5GT***	**DNA**	35,448	2,555	12,798	11,604	-	-	24,402	68.8%	12,798	11,604	-	-	68.8%
**WT *Gt3'GT***	**DNA**	29,916	2,662	5,666	5,419	5,825	4,852	21,762	72.7%	5,666	5,419	5,825	4,852	72.7%
**WT *Gt5/3'AT***	**DNA**	44,435	3,535	15,403	18,258	-	-	33,661	75.8%	15,403	18,258	-	-	75.8%
**WT *Gt5GT***	**RNA**	88,071	2,770	50,836	26,024	-	-	76,860	87.3%	50,836	26,024	-	-	87.3%
**WT *Gt3'GT***	**RNA**	62,487	2,401	4,852	2,055	28,560	22,326	31,251	92.5%	4,852	2,055	28,560	22,326	92.5%
**WT *Gt5/3'AT***	**RNA**	47,659	3,678	12,453	20,721	-	-	33,174	69.6%	12,453	20,721	-	-	69.6%

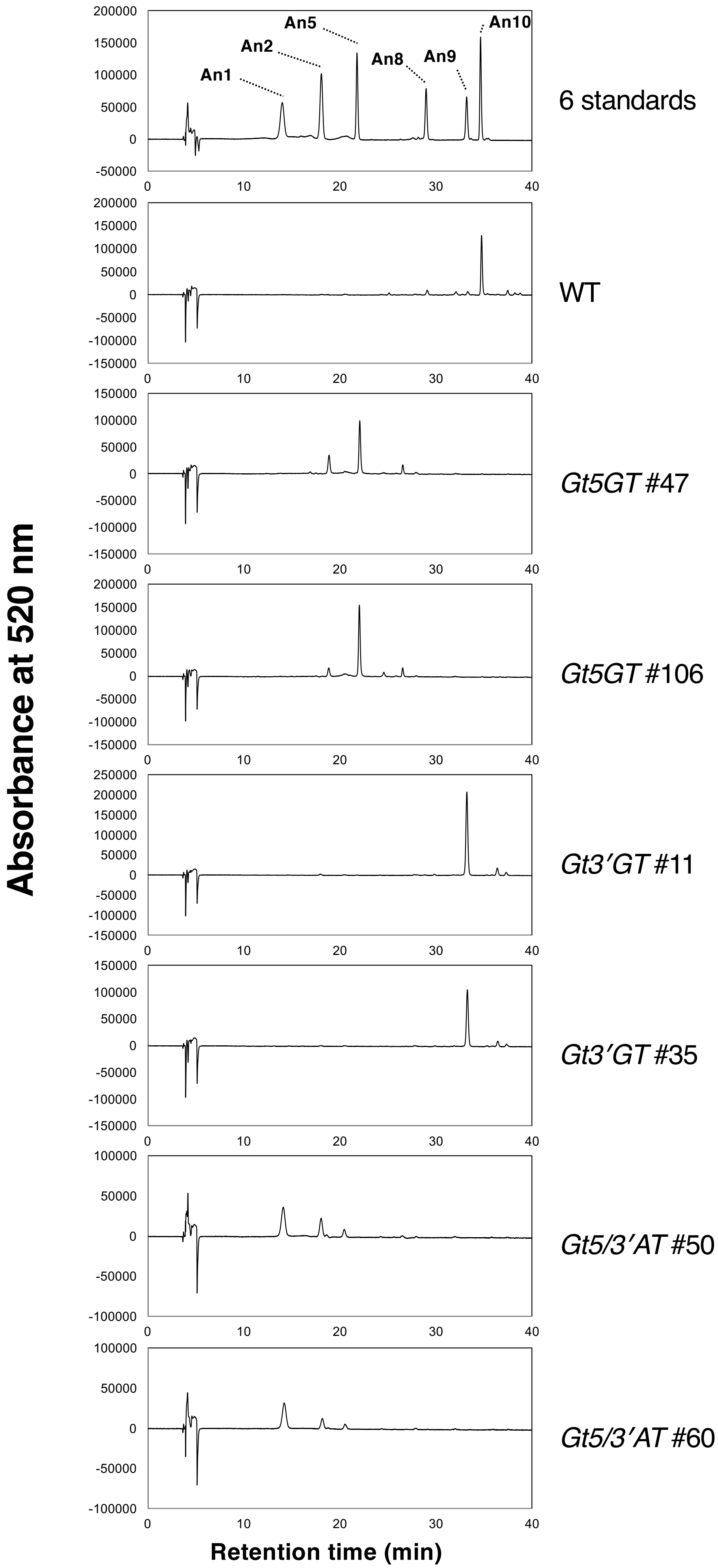

Supplementary Fig. S1 HPLC chromatograms (at 520 nm) of anthocyanins extracted from the petals of the *Gt5GT* #47 and #106, *Gt3'GT* #11 and #35, and *Gt5/3'AT* #50 and #60 lines. The WT sample and six authentic standards are the same as those described in Fig. 4.

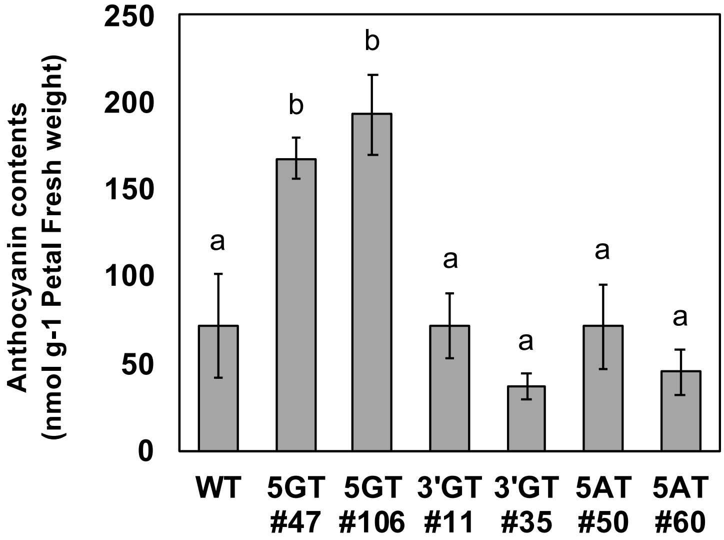

Supplementary Fig. S2

Anthocyanin concentrations in the flower petals of the genome-edited gentian plants. Quantification was performed based on the molar absorptivity of delphinidin (ε mol = 27,940 at 520 nm, evaluated in 80% MeOH containing 0.1% TFA). Vertical bars show ± SE of the means of four flowers except for *5/3'AT* #50, which was calculated using three flowers. Different letters indicate statistically significant differences in Tukey's HSD test ($P < 0.01$).

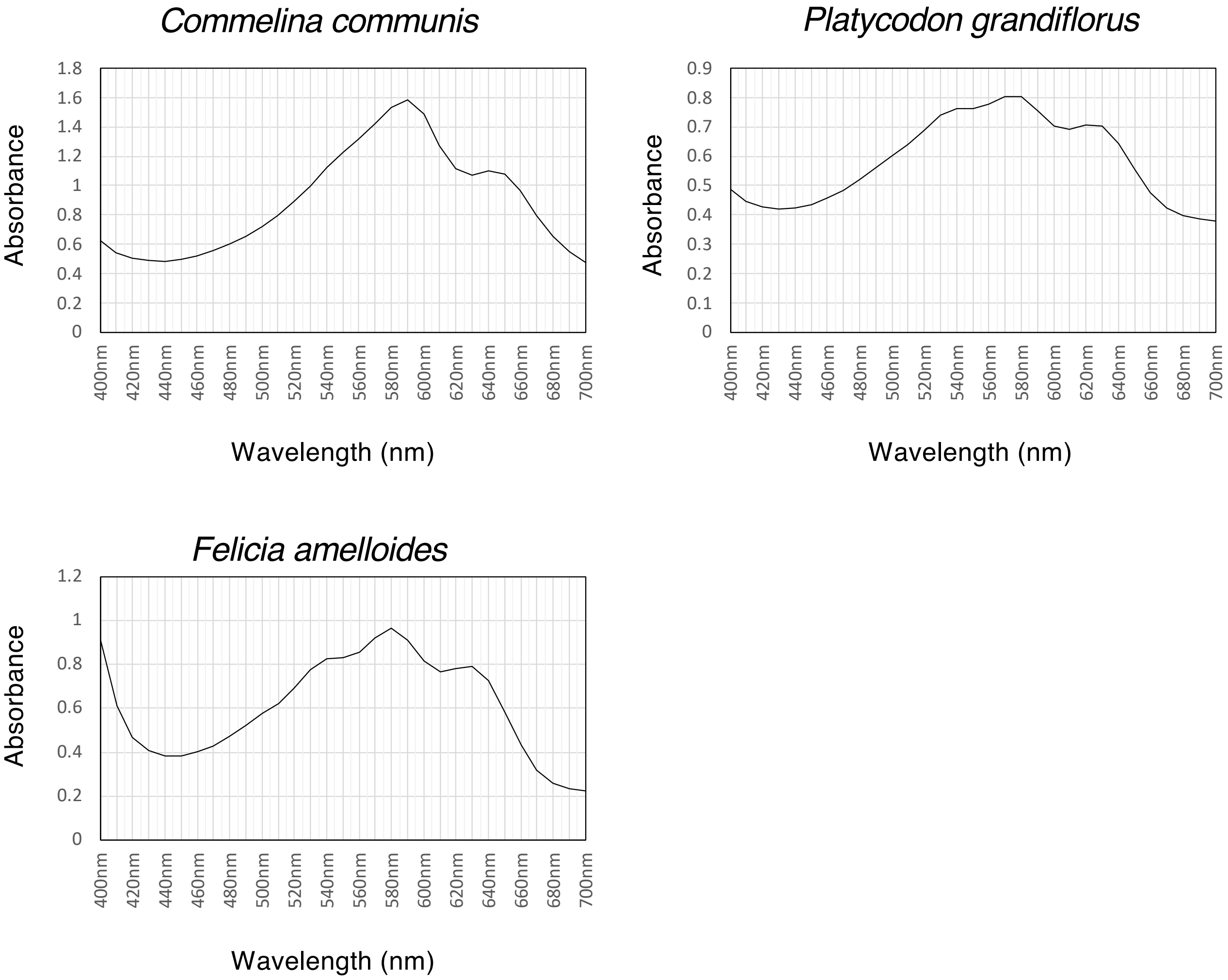

Supplementary Fig. S3

Absorption spectra of the adaxial surface of the limb area of fresh petals.

They were measured with the CM-700d spectrophotometer (Konica Minolta, Tokyo, Japan).
